# Unveiling Equine Pathologies from an Iron Age Ritual Deposit: Health, Management and Biomechanical Stress in the Protohistoric Horses of Casas del Turuñuelo (Badajoz, Spain)

**DOI:** 10.3390/ani16193058

**Published:** 2026-09-28

**Authors:** Silvia Albizuri, M. Pilar Iborra, Rafael Martínez-Valle, Joaquín Jiménez-Fragoso, Ana Isabel Mayoral, Sebastián Celestino, Jaime Lira-Garrido, Rafael M. Martínez-Sánchez, Ariadna Nieto-Espinet, Esther Rodríguez-González, Silvia Valenzuela-Lamas, Francisco J. Cambero, María Martín-Cuervo

**Affiliations:** 1 Departament d’Història i Arqueologia–SERP, Institut d’Arqueologia (IAUB), Universitat de Barcelona, 08001 Barcelona, Spain; 2 Independent Researcher, 46010 Valencia, Spain; 3 Secció d’Arqueologia, Institut Valencià de Conservació, Restauració i Investigació (IVCR+i), 46018 Valencia, Spain; 4 Grupo de Investigación Medicina y Cirugía Animal (MECIAN), Animal Medicine Department, Faculty of Veterinary, University of Extremadura, 10005 Cáceres, Spain; 5 Departamento de Medicina Animal, Facultad de Veterinaria, Universidad de Extremadura, 10005 Cáceres, Spain; 6 Instituto de Arqueología, Consejo Superior de Investigaciones Científicas-Junta de Extremadura, 06800 Mérida, Spainesther.rodrigez@iam.csic.es (E.R.-G.); 7 Área de Didactica de las Ciencias Experimentales, Dpto de Didáctica de la Matemática, Didáctica de las Ciencias Sociales y de las Ciencias Experimentales, Facultad de Ciencias de la Educación, Universidad de Málaga, 29010 Málaga, Spain; jaime.e.lira@uma.es; 8 Departamento de Historia (Área de Prehistoria), Facultad de Filosofía y Letras, Universidad de Córdoba, 14071 Córdoba, Spain; 9 Grup d’Investigació Prehistòrica (GIP), ARQHISTEC (2021-SGR-1607), Departament de Geografia, Història i Història de l’Art, Universitat de Lleida, 25003 Lleida, Spain; 10 Interaccions Medi, Bioarqueologia i Societat (IMBAS), Institución Milá y Fontanals de Investigación en Hu-manidades, Consejo Superior de Investigaciones Científicas (IMF-CSIC), 08001 Barcelona, Spain

**Keywords:** equid palaeopathology, Iberian Iron Age, Tartessian culture, Casas del Turuñuelo, biomechanical stress, enamel hypoplasia, spondylosis deformans, equid management, computed tomography

## Abstract

Equids played a central economic and symbolic role in Iberian Iron Age society, yet little is known about their health and management during the protohistoric period. This study examines the unusually well-preserved equid assemblage from the Tartessian site of Casas del Turuñuelo (Badajoz, Spain), dating from the 5th century BCE with the aim of reconstructing patterns of pathology and biomechanical stress. Thirty-one individuals were subjected to macroscopic paleopathological assessment following established diagnostic criteria and, for a subset of seven individuals, computed tomography (CT) of the thoracic and lumbar vertebrae was performed. The results reveal a consistent combination of enamel hypoplasia, abnormal wear of the second premolars, and hyperostosis of the diastema, indicating episodes of systemic stress and chronic mechanical irritation, likely related to bitting or other handling practices. Thoracolumbar spondylosis deformans and enthesopathic changes in the limbs point to repetitive strain and axial overloading compatible with traction or intensive work. Occasional metapodial synostoses further support cumulative biomechanical stress in several individuals. Altogether, these findings suggest that the horses deposited in the ritual event had undergone prolonged and sometimes demanding use prior to death. The assemblage provides rare early evidence of equine workload, welfare conditions and husbandry strategies in the southwestern Iberian Protohistory.

## Simple Summary

The protohistoric site of Casas del Turuñuelo (Badajoz, Spain) has yielded an exceptional assemblage of 41 equids deposited during a large ritual event in the 5th century BCE. Their skeletons are remarkably well preserved, allowing a detailed assessment of the animals’ health, management and life histories shortly before their sacrifice. Our study reveals a combination of dental, vertebral and limb pathologies reflecting both physiological stress and intensive mechanical loading. Enamel hypoplasia, abnormal wear of the second premolars, and hyperostosis in the diastema suggest episodes of systemic stress during development, together with chronic irritation likely caused by the use of bits or other equipment. Thoracolumbar spondylosis deformans and limb enthesopathies, in turn, reflect repetitive strain consistent with traction or heavy workload. Together, these lesions suggest that the horses were regularly subjected to demanding tasks before being sacrificed. Casas del Turuñuelo therefore offers one of the earliest and most informative windows into equine management practices in protohistoric Iberia.

## 1. Introduction

Equids occupied a central role in the socio-economic and ritual spheres of protohistoric societies and paleopathological research has enhanced our understanding of their evolution and the relationship between equids and humans. During the Early-Middle Iron Age horizon (8th–5th centuries BCE), horses acquired growing symbolic and practical importance, as reflected in artistic representations, burial practices and evidence of selective management [1]. Despite this relevance, bioarchaeological information regarding the health, husbandry and biomechanical stress experienced by these animals in the Iberian Peninsula remains scarce [2,3].

The protohistoric site of Casas del Turuñuelo (Guareña, Badajoz), which belongs culturally to the Tartessian horizon [4], provides an exceptional opportunity to address these issues due to the remarkable preservation of a large assemblage of articulated equids slaughtered as part of complex ritual events dated to the second half of the 5th century BCE [5,6]. The equids recovered at Casas del Turuñuelo correspond to at least 41 individuals in varying states of articulation and ontogenetic development. Their deposition forms part of a large sacrificial assemblage associated with the destruction of the building, offering an unparalleled window into animal management practices prior to death. The palaeopathological signatures provide key information on the nutritional status, biomechanical stress, disease prevalence, and overall life history of horses and humans. Moreover, given their chronological antiquity—ca. 2500 years old—and the uniqueness of the assemblage, these remains provide the earliest regional evidence for several equine pathological conditions.

The application of diagnostic imaging techniques to archaeological bone has transformed our capacity to document, interpret and preserve skeletal pathologies in both humans and animals. Among these techniques, computed tomography (CT)—including medical CT, high-resolution CT (HR-CT), micro-CT, synchrotron CT, and neutron/X-ray tomographic approaches—now occupies a central position because it allows non-destructive, three-dimensional visualization of internal architecture, surpassing the limitations of traditional macroscopic examination [7]. Early work in archaeology largely relied on plain radiography, which has been used since the late nineteenth century to examine Egyptian mummies and detect spinal osteoarthritis, growth arrest lines, post-mortem fractures, and dental disease. These pioneering studies demonstrated the value of imaging for distinguishing ante-mortem pathology from post-mortem alteration—an issue that remains critical in zooarchaeology and palaeopathology [8,9].

From the 1970s onward, CT revolutionized anthropological and archaeological research by enabling researchers to examine internal hard-tissue structures in unparalleled detail while leaving fragile specimens intact [10,11]. In palaeoanthropology, CT systems have been widely applied to fossil hominins and non-human primates to investigate trabecular architecture, cranial morphology, and microstructural variation, providing a methodological foundation for what is now termed “virtual anthropology” [12]. These developments are directly relevant to archaeological studies of skeletal pathology because they demonstrate how cross-sectional imaging can detect subtle internal features—such as early cortical thickening, internal bridging, or trabecular reorganization—that are often invisible on external surfaces.

Following the approach commonly applied in zooarchaeological and palaeopathological studies, this work aims to integrate macroscopic osteological evaluation with advanced imaging techniques, specifically computed tomography (CT), in order to improve the identification and characterization of pathological changes and to reconstruct patterns of animal use, welfare conditions, and management strategies within a major protohistoric settlement of southwestern Iberia.

## 2. Materials and Methods

### 2.1. Archaeological Context and Sample Composition

The skeletal remains under study come from the animal deposit found in the large open courtyard of the monumental building at the Tartessian site of Casas del Turuñuelo (Guareña, Badajoz, Spain) (Figure 1). It has been interpreted as part of the ritual activity associated with the deliberate destruction of the building and its covering with a large mound of sand. A total of 41 equids were selected, mainly males aged between 5 and 7 years, plus one female donkey, which were carefully arranged, nine of them with their bits, during three different deposition episodes. They were accompanied by a large assemblage of faunal remains, including six cattle (*Bos taurus*), four sows (*Sus domesticus*) and one dog (*Canis familiaris*) (Figure 2). Subsequently, fires and collapses occurred in the building, and it was sealed with a mound during the second half of the 5th century BCE. These fires caused damage to the bones of many animals. Furthermore, following the first phase of the animal deposit, the remains of nine equids located in the south-east corner (Figure 2) were dismembered; this has been detected both by marks left by carnivores and scavengers on their bones and by the anatomical disarticulation of the skeletons [5,6].

A total of 31 individuals with sufficient anatomical integrity and anatomical connections were selected from the group of equids for the osteopathological study (Table 1). The nine individuals found in the south-east corner have not been included, as we were unable to match the nine skulls—which allowed us to identify them and estimate their sex and ages at death—with the postcranial bones. However, we should note that numerous skeletal and dental pathologies were reported in this group. This enables us to confirm that the 41 equines in the courtyard exhibited similar characteristics in terms of their ages and clinical condition. This set is dominated by adult males, confirmed through the presence of canines, pelvic morphology and the results of the published genetic analysis. The morphometric study has made it possible to identify a large majority of horses (*Equus caballus*, *Eq ca*) and a single donkey (*Equus asinus*, *Eq as*) [5], a fact that has subsequently been corroborated by genetic studies in a total of 12 horses [13] and one donkey (Table 1, EQCA, when suitable). The height at the withers has been approximated as follows: horses: 129–140 cm; donkey: ~110 cm [5].

### 2.2. Recording Procedures and Anatomical Assessment

The analysis followed standard protocols in zooarchaeology and veterinary palaeopathology. Comparative reference material, modern veterinary descriptions, and relevant palaeopathological benchmarks were used to differentiate pathological changes from taphonomic or post-depositional modifications. Each element of Table 1 was examined macroscopically under natural and artificial light, recording:

- Indicators of the stage of development (age at death in years). Table 1 sets out the age based on epiphyseal fusion and tooth eruption/wear of the incisors [15,16,17]. It also shows the age based on crown wear [14] and cement chronology [6].
- non-pathological indicators of animal use (objects in the mouth and cheekpieces)
- dental pathology (enamel defects, wear patterns)
- evidence of biomechanical stress or repetitive strain (enthesopathies, hyperostosis, articular remodeling)

Osteological nomenclature follows veterinary anatomical standards. Photographs and detailed descriptions were produced for all pathological specimens.

### 2.3. Palaeopathological Criteria

Carbonization, thermal fracturing and deformations due to sediment collapse are present but do not prevent the palaeopathological evaluation of a large part of the affected elements. No carnivore marks or disarticulation by scavenging were observed in the best-preserved assemblages.

Lesions were classified according to established diagnostic criteria:

- Enamel hypoplasia in cheek teeth was identified by linear defects, pits, or irregular thinning of the enamel band [18,19].
- Abnormal wear (cupping, step lesions, diastema-associated wear) was assessed using comparative dental morphology [20,21].
- Hyperostosis and remodeling of the diastema, mandible and maxilla were recorded as indicators of soft-tissue inflammation or mechanical irritation [20].
- Spondylosis deformans was identified by the presence of ventrolateral osteophytes and bridging ossification on thoracic and lumbar vertebrae [22,23].
- Lower-limb pathologies: Metapodial ossification (II–IV with III) [24] and carpal, tarsal, metacarpal, metatarsal and phalanx articular degeneration (eburnation, osteophyte development) [25,26] were assessed macroscopically to determine congenital versus stress-related etiology.

### 2.4. Computed Tomography (CT) Subset and Workflow

To complement macroscopic diagnosis of axial disease, we conducted a targeted CT study on thoracic and lumbar vertebrae from seven equids selected from the Casas del Turuñuelo assemblage. Scans were reviewed retrospectively using HOROS^®^ (open-source DICOM viewer) to perform 3D volume rendering (Soft window) (Figure 3) and 3D multiplanar reconstructions (MPR) (Bone window) (Figure 4). Tools included window/level adjustment, zoom, rotation, 3D scissors and Length ROI for metric annotation. This approach enabled (i) anatomical verification and numbering, (ii) detection of internal cortical thickening and early osteophyte formation not visible externally, and (iii) precise mapping of intervertebral bony bridges.

### 2.5. Lesion Grading and Topographic Coding (CT)

For each vertebral body, osteophytic growth was graded by maximum thickness measured from original cortex to osteophyte tip (Figure 5): G0 (absent), G1 (1–3 mm), G2 (3–6 mm), G3 (>6 mm). Lesion location codes were recorded as V (ventral), VI (ventro-left), VD (ventro-right), VB (ventro-bilateral), UI/UD (unilateral), and B (bilateral). Intervertebral bridges were scored for presence/absence, grade and side. These conventions facilitated synthesis across individuals and levels and the construction of per-animal and pooled charts.

### 2.6. Criteria for Spondylosis vs. Diffuse Idiopathic Skeletal Hyperostosis (DISH)

We applied standard palaeopathological and radiological criteria. Spondylosis deformans was diagnosed by marginal osteophytes and non-flowing ventral bridging. DISH was considered when flowing ossification/bridging extended over multiple contiguous vertebrae with relative preservation of disc height and apophyseal joints, following Resnick & Niwayama for human radiology [27] and Morgan et al. for dogs (used here due to the lack of equine-specific consensus) [28], acknowledging known comorbidity between entities. For statistical tallies, DISH required flowing bridges across ≥3 contiguous vertebral levels in line with veterinary adaptations.

### 2.7. Sample Notes and Limitations (CT)

One equid with extensive loss of critical levels was excluded from DISH tallies but described qualitatively, yielding six equids in pooled CT statistics. We note that taphonomic loss or fragmentation occasionally required inferential assessment of former bridging where complementary counterparts were missing, and extraspinal manifestations could not be evaluated in this dataset.

## 3. Results

### 3.1. Overview of the Assemblage

In the total number of complete equids studied—that is, 31 specimens—pathologies were observed that extensively affected the skeleton, although the extent varied from one case to another. Most animals showed signs of damage caused by the use of a bit, even though only nine still had it in situ, in their mouths or cheeks. However, not all of them showed damage to both the limb bones and the spine. Finally, all 31 individuals showed signs of hypoplasia in their teeth. These pathologies were found in both the youngest animals, aged between 2 and 4 years, and the adults (aged between 4 and 9 years) (Table 1).

Most of the animals were between 5 and 7 years old; one was sub-adult, a few were between 4 and 5 years and others lived beyond the age of 7, up to the age of 9. These pathologies are concentrated in the vertebral column and the appendicular skeleton and the mouth (mainly wear and damage caused by biting and by nutritional deficiencies—hypoplasia) (Figure 6). Furthermore, sharp enamel points have been observed in the enamel of some horses, such as lateral ridges on the maxillary teeth, medial ridges on the mandibular teeth, or sharp focal points at the mesial and distal ends of the dental arches (Figure 7).

In equids, age at death is determined based on the degree of eruption and wear of the incisors and on epiphyseal fusion. Cementochronology and crown height have also been included for comparison with estimates derived from bone maturity and incisor wear. In general, the estimated ages are similar in all cases, although they are slightly higher when measured from the crown, particularly in some individuals over 5–6 years of age (Table 1), which could be linked to wear and shortening of the crown caused by a diet containing abrasive components [29,30].

Adult individuals aged between 4 and 5 years (*n* = 4; see Table 1) are characterized by epiphyseal fusion of the postcranial skeleton and juvenile dentition (unworn incisors and canines). However, the epiphyses of the vertebral bodies of some thoracic vertebrae appear to be in the process of fusion, as is also the case in some equids aged between 5 and 6–7 years, such as EQ9 and EQ10 (Figure 8). The age at which the epiphyses fuse varies among modern breeds, occurring between 3 and 4 years of age [31], and occurs later in Thoroughbred racehorses, between 4.9 and 6.7 years [32]. The latter exhibit the same pattern observed at the Casas del Turuñuelo site.

### 3.2. Dental and Oral Pathologies

Enamel defects consistent with hypoplasia were observed in the lower and upper jaws of 100% of the 31 animals studied. These are mainly characterized by linear grooves and are located on the lingual and labial surfaces of the enamel of the premolars and molars. (Figure 9).

Abnormal wear patterns not related to malocclusion were observed in 80.6% of cases affecting the upper and lower second premolars (PM2).

Two types of wear marks are evident on the anterior edge and occlusal surface of the second premolars. In both types, asymmetries are observed between the left and right sides of the mouth:

1. Slight wear with enamel exposure on the lower premolars (Figure 10A).
2. Deep bevel wear on the upper and lower premolars, with exposure of the enamel and dentine, resulting in V-shaped occlusal wear with a more or less pronounced angle (80.6%) (Figure 10B,C).

Both wear types are compatible with metal bits and cheekpieces found in the mouths of nine animals (Table 1; Figure 10D,E).

Additionally, 35.5% of the specimens showed hyperostosis/reactive remodeling in the diastema, interpreted as a response to chronic mechanical irritation or inflammation of adjacent soft tissues (Figure 11), related to the use of bits. Among them was the donkey EQ20, which had retained canines (Figure 11), indicating that this animal had also been bit-trained.

### 3.3. Appendicular Skeleton: Stress and Congenital/Degenerative Change

Within the appendicular elements, we documented: enthesopathy changes and marginal remodelling at the tendon and ligament insertions, with abnormalities in the metapodial bones, including synostosis or fusion of metacarpal elements accessory to the main axis (66.7%) and osteoarthritis of the phalanges (40,7%).

Osteophytes have been observed at the posterior or palmar ends of the proximal phalanges of the forelimb (anterior), at the point of insertion of the flexor tendons. However, in some cases, it affects the hindlimb (posterior) and the intermediate phalanges (Figure 12B,C).

Enthesopathic changes affect the joint areas of the carpus and tarsus, and are sometimes also observed in the proximal region of the metatarsus (Figure 12A) and phalanx (Figure 12B–D). Fusion of the lateral metapodials has only been observed in the metacarpals and enthesopathic changes have also been observed (Figure 13).

### 3.4. Axial Skeleton: Vertebral Disease

In the thoracolumbar column, multiple vertebrae presented ventrolateral exostosis with partial to complete bone bridging formation, diagnostic of spondylosis deformans, in 88.5% of the equids (Figure 6, Figure 14A,B, Figure 15 and Figure 16).

In two cases, EQ45 and EQ61, the transverse, highly demarcated linear radiolucency observed on the anterior aspect of the vertebral body (Figure 14C) is a well-documented anatomical variant known as a persistent Hahn’s cleft (or Hahn’s canal). In bioarchaeological and forensic contexts, this manifestation is classified as a non-metric epigenetic trait rather than a pathological lesion. A persistent Hahn’s cleft occurs when the horizontal neurovascular bundle, specifically the basivertebral veins and nutrient arteries, prevents the complete equatorial fusion of the primary superior and inferior ossification centres of the foetal vertebral body. As ontogenetic development progresses, this incomplete osseous bridging leaves a distinct, non-pathological horizontal cortical groove along the midline of the bone.

### 3.5. CT-Based Discrimination of Axial Entities

Tomography resolves several ambiguities inherent to macroscopic inspection alone (Figure 17A,B). In our subset, all scanned equids exhibited spondylotic change, while half displayed DISH-compatible flowing bridges that met adapted radiological thresholds. Importantly, CT revealed internal cortical thickening and early ventral osteophytes in segments that appeared unremarkable externally, underscoring the value of cross-sectional imaging for identifying incipient disease. The coexistence of spondylosis and DISH within individual mirrors patterns reported in clinical veterinary radiology and palaeopathology, advocating for explicit dual coding rather than mutual exclusion in archaeological diagnoses.

### 3.6. Regional Patterning and Lateralization

CT shows that bridging concentrates in caudal thoracic and cranial lumbar levels, consistent with zones of peak mobility transition and load transfer along the equine torso (Figure 18). Within these regions, higher-grade lesions (G3) cluster in late thoracic; lower-grade osteophytes occur more cranially, and several individuals exhibit left-sided or ventro-left bias in advanced grades—patterns compatible with asymmetric loading, equipment pressure or directional tasks (Figure 19).

## 4. Discussion

Paleopathological analysis of skeletal remains recovered from archaeological contexts provides valuable insights into the lifeways of past societies that coexisted with and managed animals [21,22].

Initial assessments suggest that these animals, including sub-adult horses, adults and the elderly, all male and a female donkey, display a wide spectrum of skeletal and dental pathologies, such as abnormal premolar wear, hyperostosis and remodeling in the diastema, and vertebral alterations compatible with spondylosis deformans. Additional lesions, such as fusion of metapodials (congenital or stress-related synostosis) and alterations in distal limb bones, reveal patterns consistent with mechanical overload and repetitive strain, likely associated with traction or early training. The co-occurrence of enamel hypoplasia and abnormal PM2 wear with diastema hyperostosis points to combined physiological and mechanical stressors.

### 4.1. Dental Findings

Episodes of linear hypoplasia are consistent with systemic stress during amelogenesis (e.g., nutritional deficits, disease, or problematic weaning) at the time of tooth formation [18,19]. Paleoclimatic reconstructions of the south-west of the Iberian Peninsula indicate that, around 2500 years ago, during the Middle and Late Iron Age, climatic conditions were slightly warmer and drier than they are today, with moderate aridity manifested in greater summer dryness [33]. Isotopic analyses of the horses’ tooth enamel suggest that their diet was supplemented, in some cases and during the summer, with cereal-based fodder [33], which could explain the appearance of linear grooves caused by nutritional deficiencies during dry periods. Fodder containing straw is less nutritious and more abrasive, which may explain the nutritional deficiencies and the reduction in crown height observed in some individuals during cementochronological analysis, whilst not ruling out a natural diet on siliceous soils. Furthermore, it is worth noting that numerous charred seeds have been found amongst the animals from the Casas del Turuñuelo enclosure, with barley seeds being particularly abundant [34], suggesting the use of grain as horse feed. This may be linked to the ‘sharp enamel points’ detected in some animals, as feed containing grain causes uneven wear on the occlusal surface and results in sharp points [29,30].

The pattern of focal wear on PM2 and diastema hyperostosis is consistent with chronic bit/tackle irritation and repeated microtrauma when horses are employed under intensive management.

The wear observed on the PM2s from Casas del Turuñuelo comprises two types: slight wear on the lower PM2 teeth, consistent with the use of metal bits and bridles, which are found in situ on some animals. Deep, beveled wear marks are also observed on the lower and upper PM2, consistent with the driving of carts [21]. V-shaped occlusal wear has been observed in Mongolian horses in Mongolian riding horses [35] and has also been confirmed in a horse from Illerup Ådal (Denmark) dating from the 3rd century AD [36]. This wear coincides with the position of a metal bit and the pressure of the bridles on both maxillary and mandibular PM2. Carriages drawn by two horses can cause uneven pressure and asymmetrical wear on both sides of the animals’ cheeks, varying according to the position of each horse [37]. This has been observed in six animals from Casas del Turuñuelo, such as EQ9, which shows more pronounced wear on the maxillary and mandibular PM2 teeth on the left side (Figure 10B,C). However, it is likely that many factors influence the formation of this characteristic, including the animal’s age when the bridle was fitted, the type and fit of the bridle, and the frequency and nature of the riding or transport for which the animal is used [35]. In this regard, it is worth noting that during the Early Middle Iron Age, depictions of chariots were common on the Iberian Peninsula, but there are also records of certain chariot-related artefacts, notably the Tartessian artefact found in Tomb 17 at La Joya (Huelva), which features a yoke for two horses [38].

### 4.2. Appendicular Pathologies

Enthesopathies and appendicular remodeling reinforce the working hypothesis of traction and/or early training. Metapodial, carpal and tarsal synostoses (when present), and finally phalanx, can have dual etiologies (congenital vs. functional)**.** Their contextual reading with other stress markers suggests significant mechanical contribution in several specimens.

Fusion of the lateral metapodials has only been observed in the metacarpals. In modern horses over 2 years of age, this phenomenon is accentuated regardless of the work they perform [39]. These pathological findings are related to trauma and other factors such as age. Small lesions may also be attributed to tendon strain due to the exertion associated with pulling carts and/or ploughs [24,25]. The animals from Casas del Turuñuelo exhibiting synostosis or fusion of the metacarpals were over five years old and showed spinal pathologies and wear on the PM2; in some cases, these were associated with cheekpieces. The evidence of their use for work is therefore clear. There are, however, two exceptions: EQ12 and EQ13, aged 4 and 5 years at the time of death, which exhibit pathologies and bit-related issues associated with the animal’s early use (Table 1).

### 4.3. Spine Pathologies

Diffuse idiopathic skeletal hyperostosis (DISH) is defined as a systemic pathological condition affecting both the axial and peripheral skeleton, resulting in the ossification of soft tissues. Spinal hyperostosis, also referred to as “Forestier’s disease,” has been described in the medical literature under various denominations since 1824; however, it was not until the 1970s that Resnick and Niwayama characterized the presence of extraspinal manifestations as part of the DISH spectrum [27]. In the veterinary literature, DISH was not reported until the 1980s, initially in dogs [28]. In veterinary medicine, three types of osseous proliferative conditions that may lead to vertebral fusion are generally recognized: ankylosing spondylosis, spondylosis deformans, and DISH. Although bibliographic references to DISH in horses remain scarce, particular attention should be given to the published study addressing this condition in equine skeletal remains from the Gallo-Roman period [40]. The spondylosis deformans identified in thoracolumbar segments reflects chronic overloading of the axial skeleton. In medieval European contexts, spondylosis and other degenerative lesions of the equine musculoskeletal system have been associated with inappropriate or excessive use (e.g., draught, sustained load, hard surfaces), offering a useful comparative framework for interpreting the protohistoric cases of Casas del Turuñuelo. While aetiology is multifactorial for DISH, the tomographic distribution aligns with repetitive axial overloading inferred from macroscopic spondylosis deformans and supports interpretations of traction or intensive use prior to sacrifice.

The persistent Hahn’s clefts (or canals of Hahn) observed on the thoracic vertebrae of two equids from the archaeological site (EQ45 and EQ61) represent a benign, non-pathological developmental variant rather than a traumatic event [41,42]. In zooarchaeological analysis, this congenital manifestation must be clearly differentiated from both antemortem and taphonomic fractures based on distinct macromorphological criteria. Unlike antemortem fractures or fissures, the cleft lacks any macroscopic indicators of vital tissue response, such as subperiosteal osteogenic remodeling, reactive woven bone, macro-porosity, or callus formation, confirming that the animal did not suffer a live compressive trauma [43]. Furthermore, it is easily distinguished from taphonomic fractures caused by dry-bone weathering, excavation damage, or soil sediment pressure; taphonomic breaks characteristically present sharp, jagged, and un-remodeled internal trabecular profiles that expose a lighter bone color matrix. In contrast, these equid specimens display smooth, corticated, and completely regular margins inside a perfectly symmetrical, horizontal midline groove, which is the unequivocal signature of an incomplete ontogenetic fusion surrounding the primary basivertebral vascular tract. Levine observed this horizontal line in archaeological specimens, specifically in the fourteenth thoracic vertebra of Horse 4 from Kurgan 3 at Ak-Alakha 5 [22], as well as in free-ranging Exmoor ponies [25].

### 4.4. CT-Axial Study

CT-confirmed axial bridging integrates with the oral evidence (abnormal PM2 wear and diastema hyperostosis) and appendicular stress markers to form a coherent biomechanical narrative. As observed in comparative medieval datasets, work-related misuse/overuse preferentially targets the musculoskeletal system; in our protohistoric sample, the same mechanical logic applies, though the cultural and ritual context differs. CT thus strengthens the case that management practices—not merely ageing—drove much of the observed pathology. Thoracic and lumbar spondylosis (88.5%), metapodial exostoses and metacarpal synostosis (66.7%), and proximal phalanx osteoarthritis (40.7%) compose a stress signature consistent with repetitive axial loading and limb overuse typical of riding and/or traction. A tomographic subset of Casas del Turuñuelo equids further supports this: CT of seven thoracolumbar columns identified spondylosis in all scanned animals and DISH-compatible flowing bridges in half, corroborating cumulative mechanical stress beyond what macroscopic surfaces alone can reveal.

This study demonstrates the feasibility and interpretive payoff of retrospective CT in archaeozoology: (i) objective grading of osteophytes by thickness; (ii) confirmation of “flowing” morphology across levels; (iii) differential diagnosis between spondylosis and DISH in the absence of equine-specific criteria; and (iv) mapping of lesion sidedness that may reflect handling practices. Future work should combine CT with targeted histology and, where possible, extraspinal imaging to evaluate DISH’s systemic footprint.

### 4.5. Limitations

Absolute counts of individuals per lesion may be under/overrepresented by differential fragmentation, anatomical overlap, and thermal effects. Even so, the anatomical and topographic consistency of the lesions, their repetitive nature, and the articulated assembly mitigate taphonomic biases and support inferences about use and management. Additional imaging of appendicular and cranial regions could provide further insights into the extent and nature of skeletal pathology.

### 4.6. The Selection of Horses: Working Animals?

The distinctive feature of Casas del Turuñuelo lies in the combination of a mass ritual sacrifice involving mainly adult male horses, which are in an excellent state of anatomical preservation and show clear evidence of having been used or trained prior to their death. This allows us to reconstruct life histories in which horses were assets of great functional and symbolic value, affected by cumulative biomechanical stress and episodes of systemic stress. The correlation between oral lesions, vertebral degeneration and changes in limb overload presents an integrated pattern of intensive use and chronic overload.

The beveled wear of PM2 observed in 80.6% of individuals differs markedly from wear attributable solely to diet and strongly supports an interpretation suggesting mechanical stress related to biting. The presence of pathological conditions among the youngest animals suggests that the process of training and putting them to work began before the age of four, before they had reached a certain level of growth and maturity, which must have affected their growth. Nowadays, to avoid these problems, horses are put to work later. Furthermore, the presence of cheekpieces in nine cases, one of which was found in an animal aged between 4 and 5 years (EQ13), confirms this hypothesis.

The fact that most of the animals ranged between 5 and 7 years of age and that they were male suggests they were selected and sacrificed during their productive years, rather than due to illness or natural causes. However, the high incidence of work-related conditions may indicate that animals with reduced capabilities were used for sacrificial purposes. In Book IV of Columella’s *De re Rustica*, horses intended for domestic service must be broken in at the age of two, whilst those set aside for combat must not be broken in until they have reached the age of three; however, they must not be subjected to the rigours of combat until they have reached the age of four. Mongolian horses begin their training for riding between their first and second years of life [35].

Other pathological case studies from the European Iron Age show similarities with the horses from Casas del Turuñuelo. Studies in Bulgaria by [44] indicate that the animals underwent early training, which may have contributed to an increase in stress-related pathologies, with a pattern of death at a relatively young age, around 6 years. They demonstrate that repetitive loading of the thoracolumbar region—particularly under conditions of traction, weight-bearing and early training—led to degenerative changes and compensatory bone reactions in the spine and distal limbs. Increased stiffness of the thoracolumbar spine alters load transmission through the limbs, potentially contributing to periosteal proliferation and osteophyte formation at ligament attachment sites, including the proximal phalanges. In addition, wear on the PM2 teeth and the diastema indicates the use of metal bits for harnessing the animals for both riding and pulling carts. Osteological comparisons between riding horses and cart horses in East Asia [21] suggest that riding may cause more damage to the lower jaw, whilst use in carts may result in damage from the bit being distributed more evenly across both the upper and lower dentition. Furthermore, the burials of pairs of horses, associated with elite chariot burials at the Shinjia site, in Gansu Province, China (c. 1600–1046 BCE), show asymmetry in their bone pathologies, particularly in the diastema and the distal end of the thoracic vertebrae, with a high incidence in limb bones [37]. Although comparative studies suggest that certain pathological patterns may be associated with riding or traction activities, the skeletal evidence from Casas del Turuñuelo does not permit a secure functional attribution of individual animals. Therefore, we interpret the observed lesions as evidence of intensive use and chronic biomechanical stress rather than as indicators of a specific working role.

## 5. Conclusions

The Casas del Turuñuelo equids exhibit a coherent paleopathological signature—enamel hypoplasia, abnormal PM2 wear with diastema hyperostosis, thoracolumbar spondylosis deformans, and appendicular stress markers—indicative of intensive management, traction and early training— and episodes of systemic stress prior to their ritual deposition.

The combination of oral, axial and appendicular lesions points to a multifactorial aetiology involving mechanical stress and physiological strain, which is consistent with zooarchaeological observations suggesting that equine pathologies often reflect early and excessive use associated with riding and the pulling of carts. The high incidence of work-related conditions may indicate that animals with reduced capabilities were used for sacrificial purposes.

Casas del Turuñuelo provides one of the earliest and best-preserved datasets for equine health and management in the southwestern Iberian Protohistory, offering a benchmark for regional and supra-regional comparisons of workload, welfare, and ritual economy. Furthermore, the identification of specific lesions and treatment patterns may shed light on human–animal relationships, including care strategies, exploitation intensity, and the potential symbolic or ritual significance attributed to these animals within the community.

## Figures and Tables

**Figure 1:**
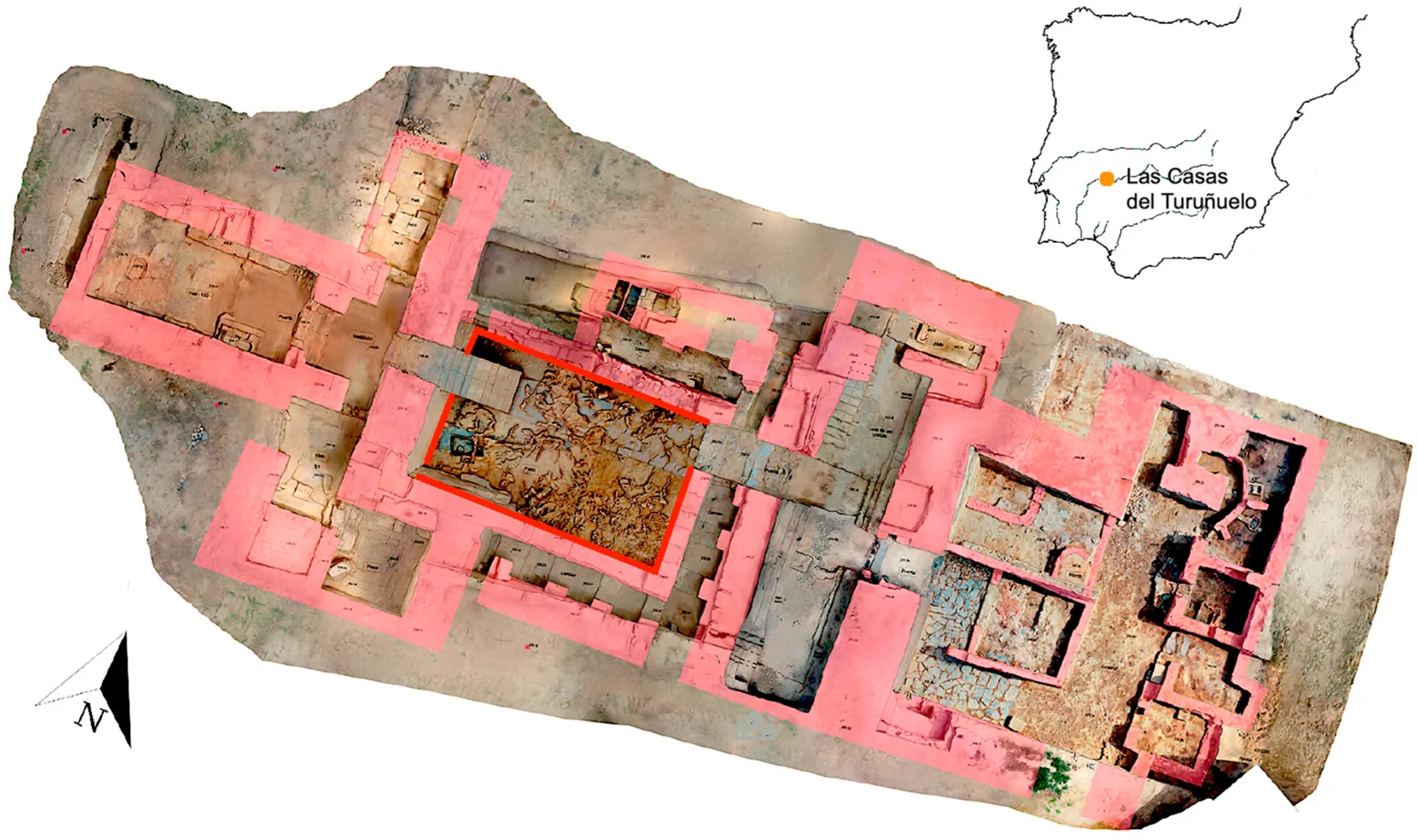
Layout of the Turuñuelo building. The courtyard is marked in red. Photogrammetry carried out in June 2024.

**Figure 2:**
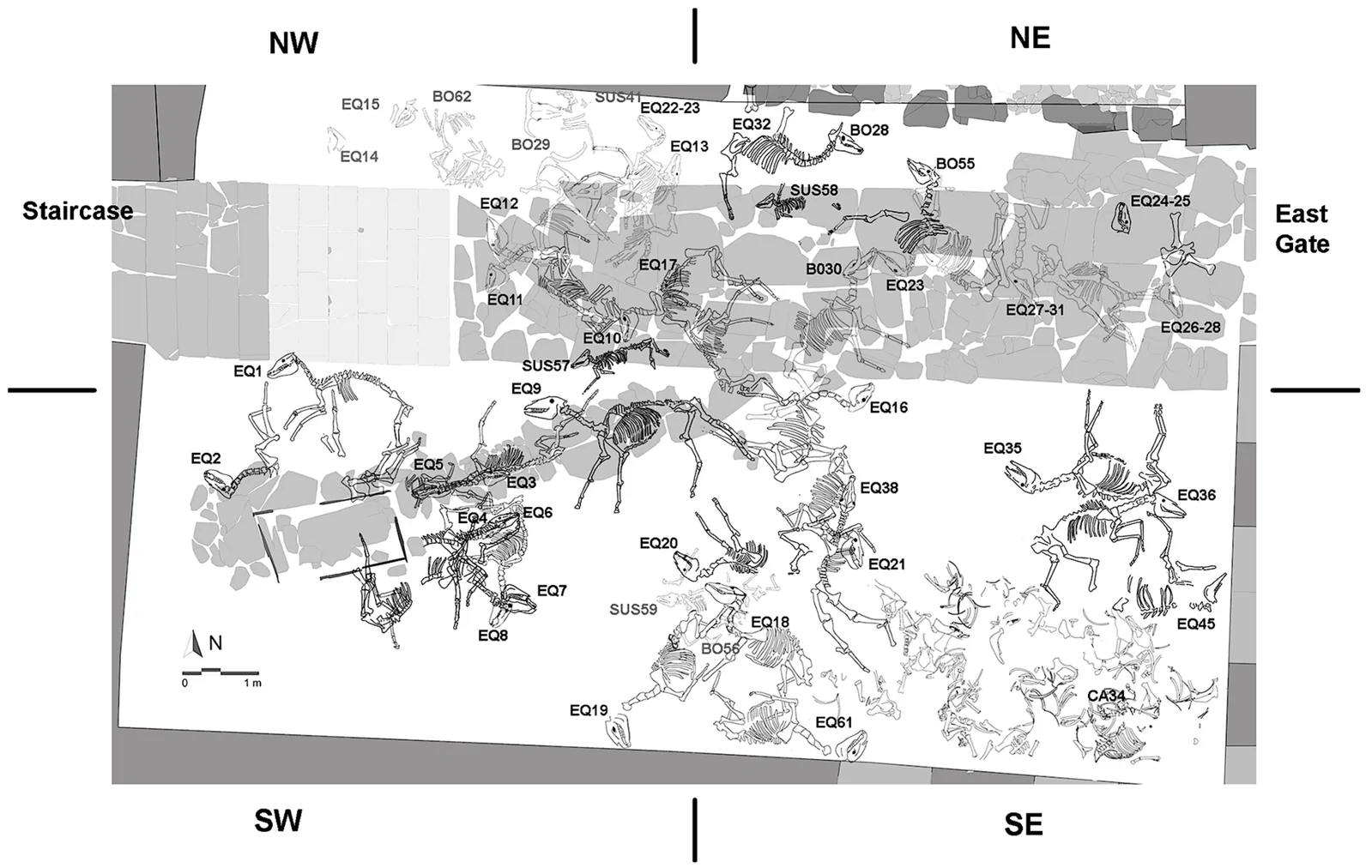
Faunal deposit from the courtyard. 32 complete equids (EQ), six cattle (BO) and four pigs (SUS). In the south-east (SE) corner, partial skeletons and isolated remains of a total of 9 equids (Minimum Number of Individuals, MNI) and those of a dog (Ca34) were recorded.

**Figure 3:**
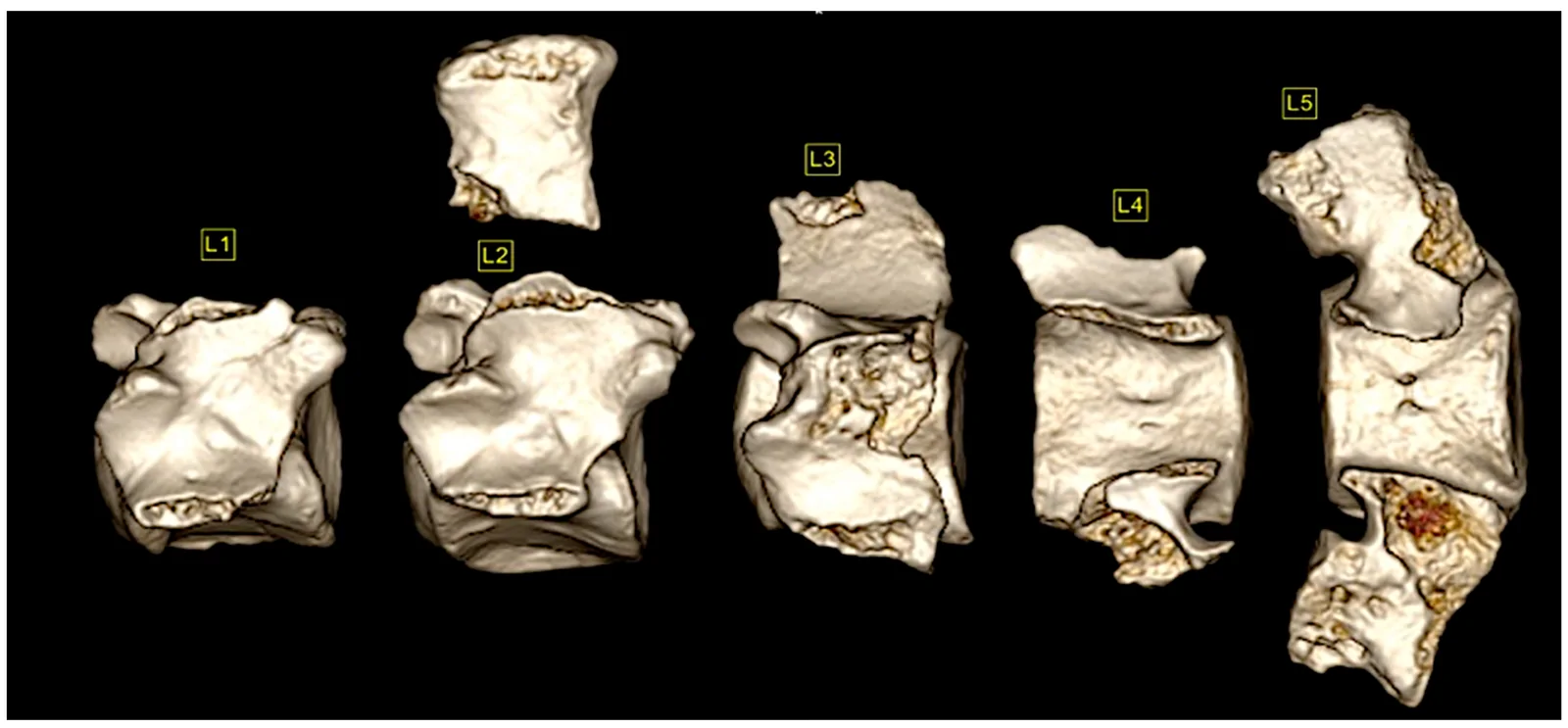
Volumetric reconstruction in HOROS^®^ Version 4.0.0 of the lumbar vertebrae (L1–L5) of one of the equids included in this work, after establishing the correspondence with the anatomical positions of each of them. The reconstruction highlights post-excavation fragmentation of the spinous process of L2, attributed to handling of the specimen. Small cortical erosions or areas of cortical loss are also visible on several transverse processes, particularly in L1, L2 and L4. In L5, bone remodelling is observed on the cranial aspect of the vertebral body, consistent with pathological new bone formation. These features can be distinguished from the taphonomic alterations affecting other anatomical regions and illustrate the preservation quality and pathological changes detectable through CT-based three-dimensional reconstruction.

**Figure 4:**
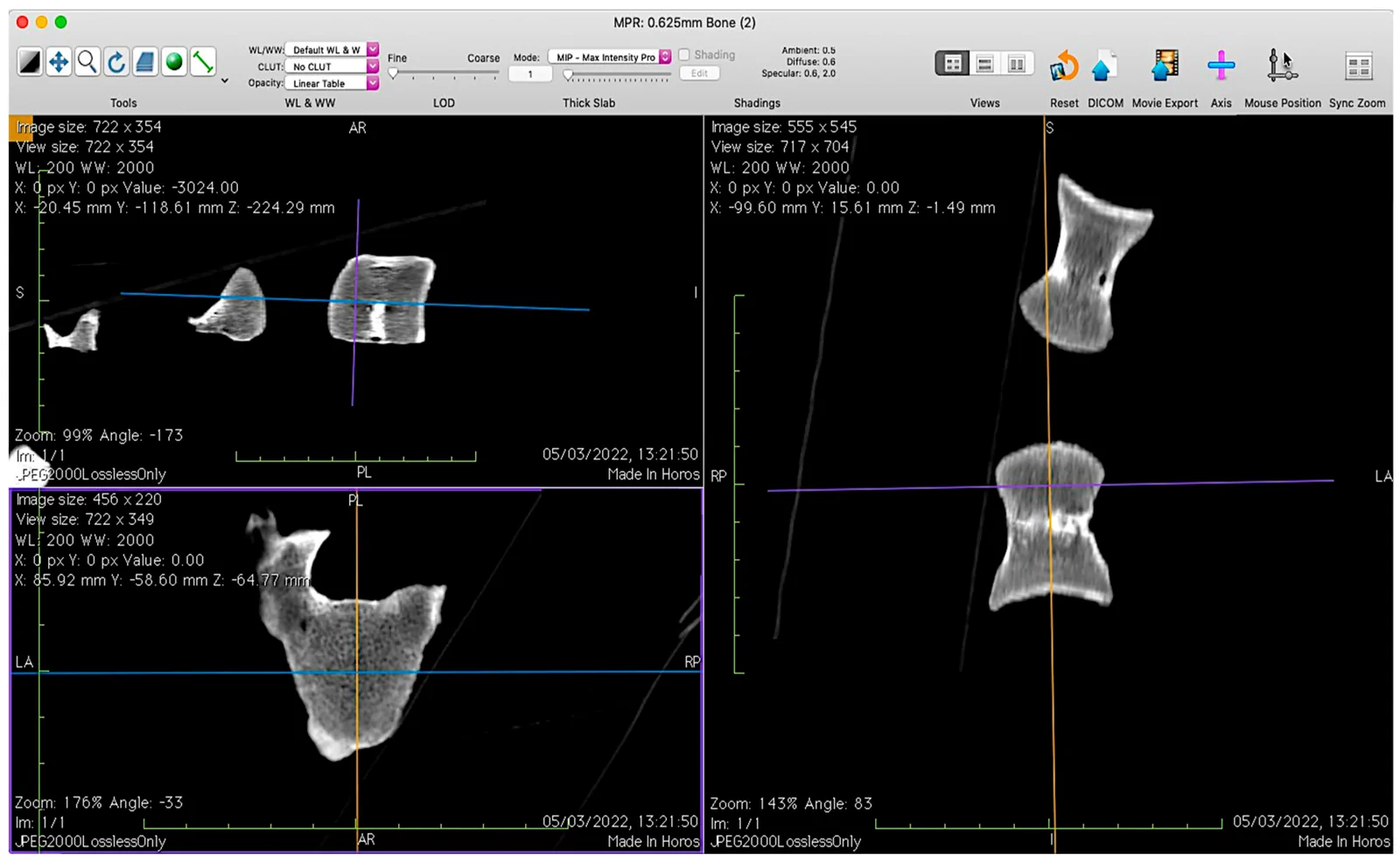
Multiplanar reconstruction in HOROS^®^. The vertebral body is observed in the three planes: sagittal (**left above**), transverse (**left below**) and dorsal (**right**). This figure illustrates the level of anatomical detail provided by CT imaging, allowing the assessment of features ranging from cortical bone thickness to subtle internal skeletal alterations that may not be detectable through macroscopic examination alone. As an example, the transverse view reveals an incipient and asymmetrical area of bone remodelling on the left ventral aspect of the vertebral body. The image highlights the value of CT−based analysis for the identification and characterization of early pathological changes and minor osseous modifications in archaeological skeletal remains.

**Figure 5:**
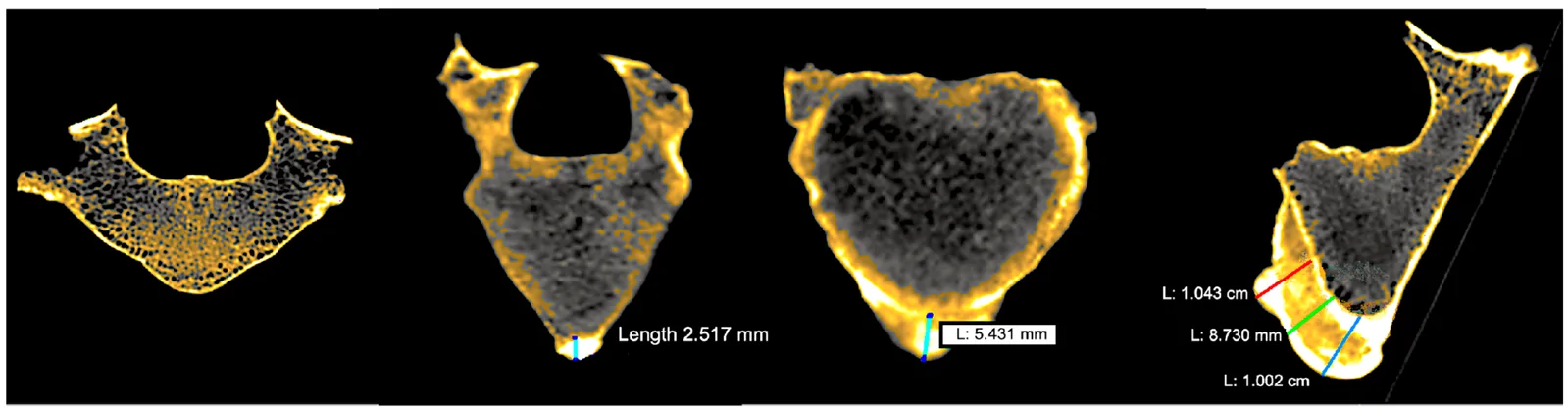
Images visualized in HOROS^®^ of vertebrae in multiplanar reconstruction that present the four degrees of ossification detailed together with their respective measurements. From left to right: G0, G1 (2.5 mm), G2 (5.4 mm) and G3 (8.7–10.4 mm).

**Figure 6:**
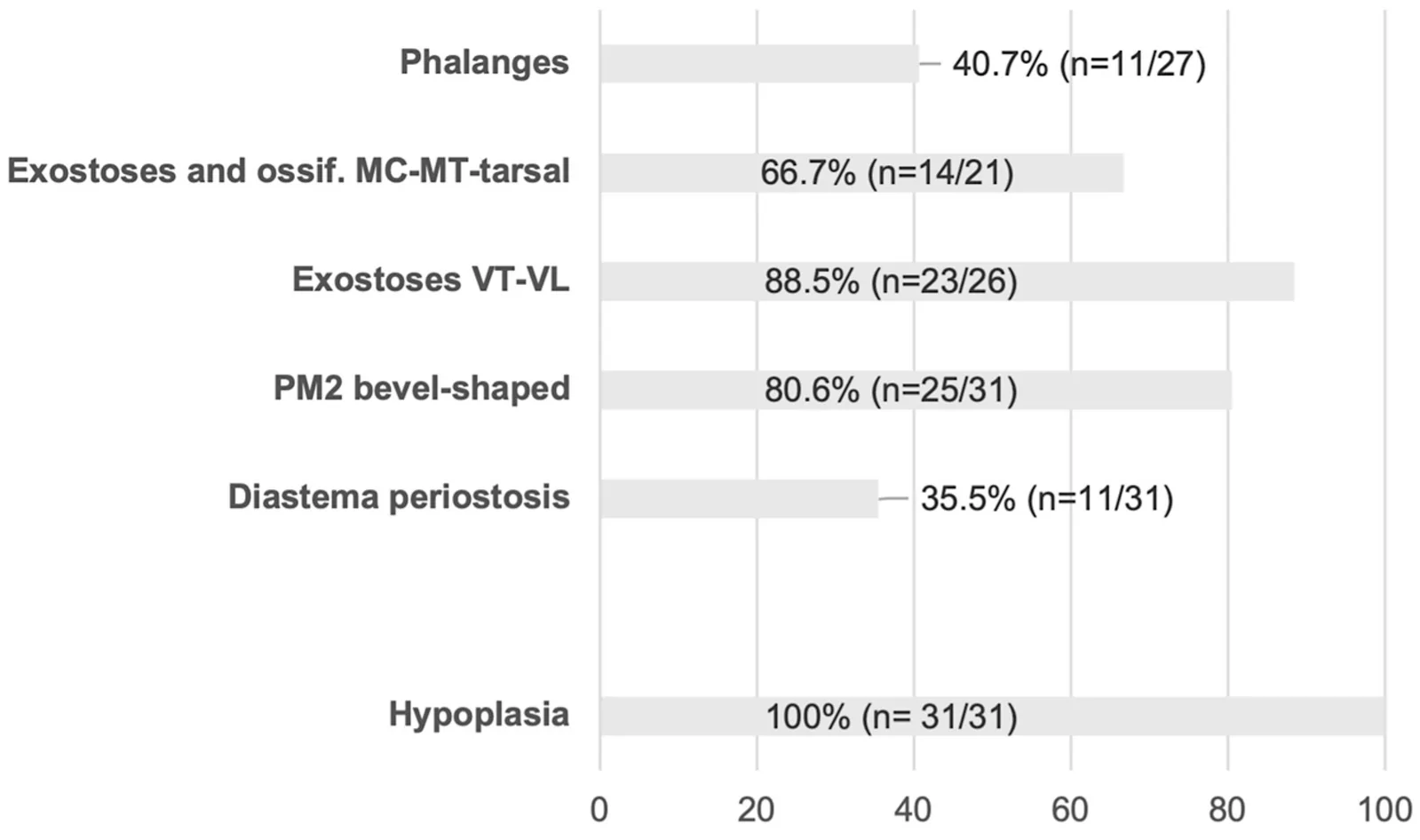
Prevalence of key macroscopic lesions. The prevalence of each lesion was calculated as a proportion of the group analyzed (*n* = x/27, 21, 26, 31). The percentages have been calculated without taking into account equines that are missing anatomical parts or are severely fragmented or burnt (shown in grey in Table 1).

**Figure 7:**
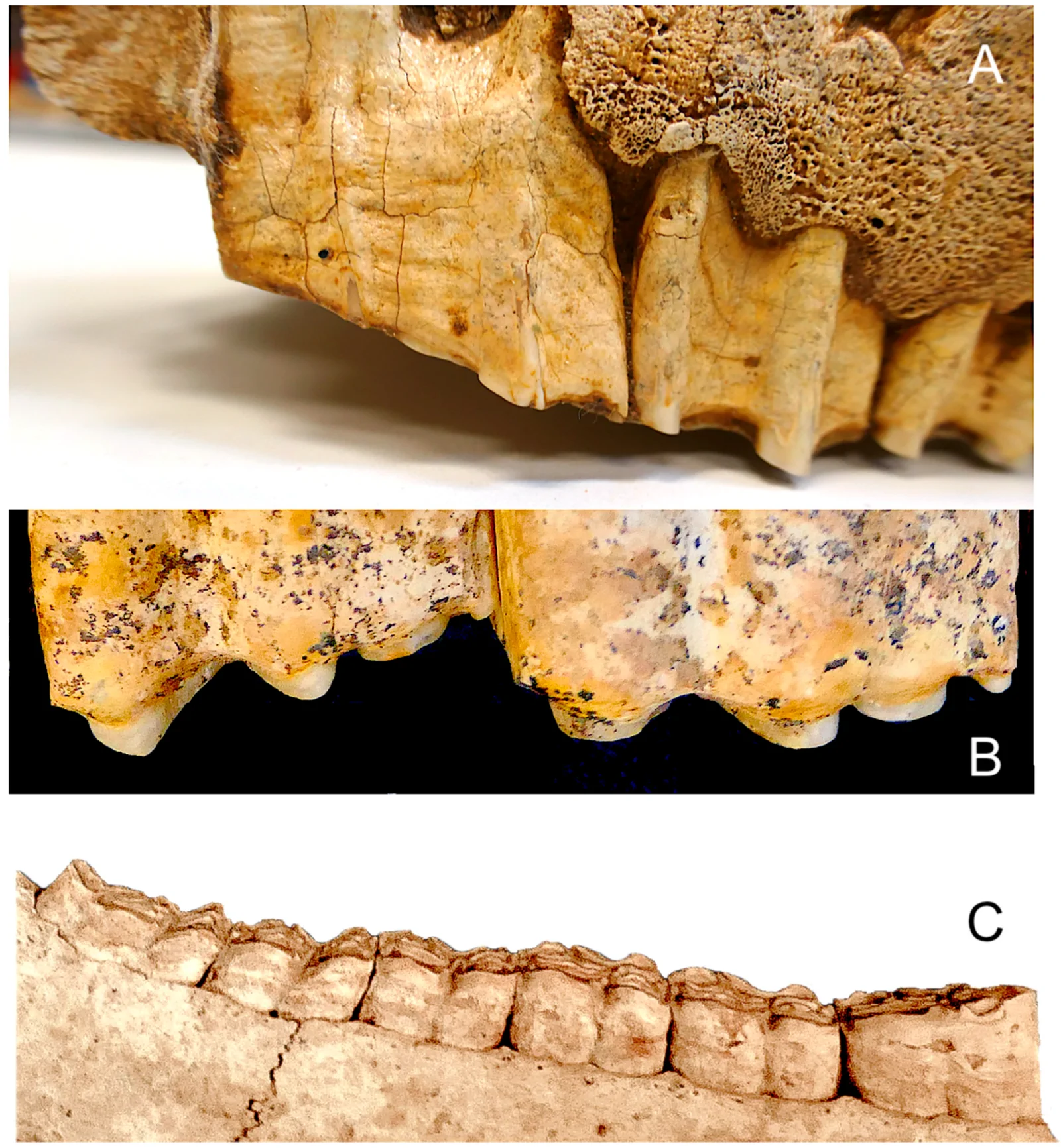
Enamel points and sharp focal points. (**A**) EQ45, enamel points in the premolar series of the left maxilla (palatal view). (**B**) EQ24–25, mandibular enamel points (vestibular view). (**C**) EQ19, sharp focal points at the mesial and distal ends of the mandibular arch (vestibular view).

**Figure 8:**
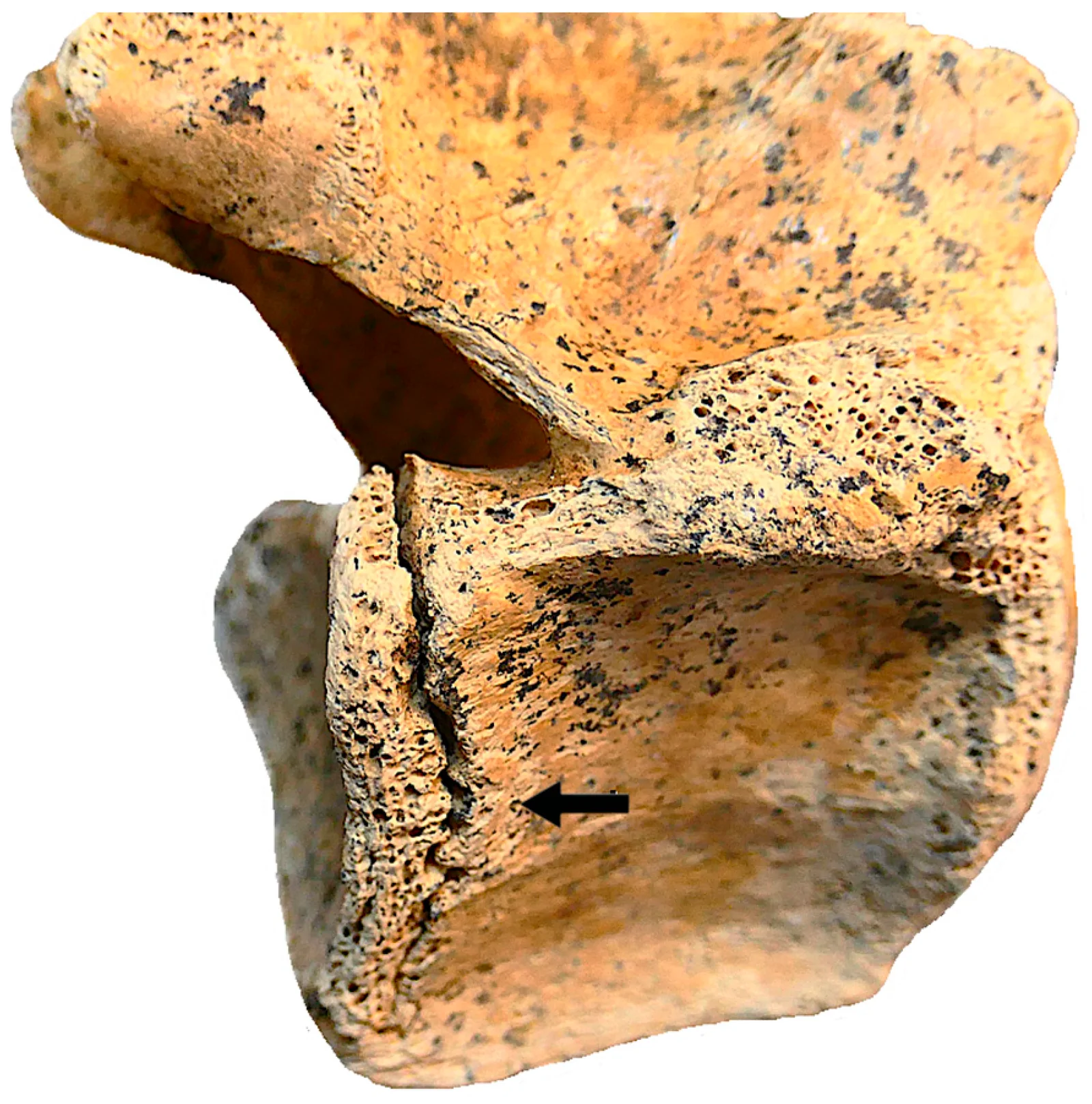
Thoracic vertebrae of specimen EQ10, showing partial fusion of the caudal epiphyses with the vertebral body.

**Figure 9:**
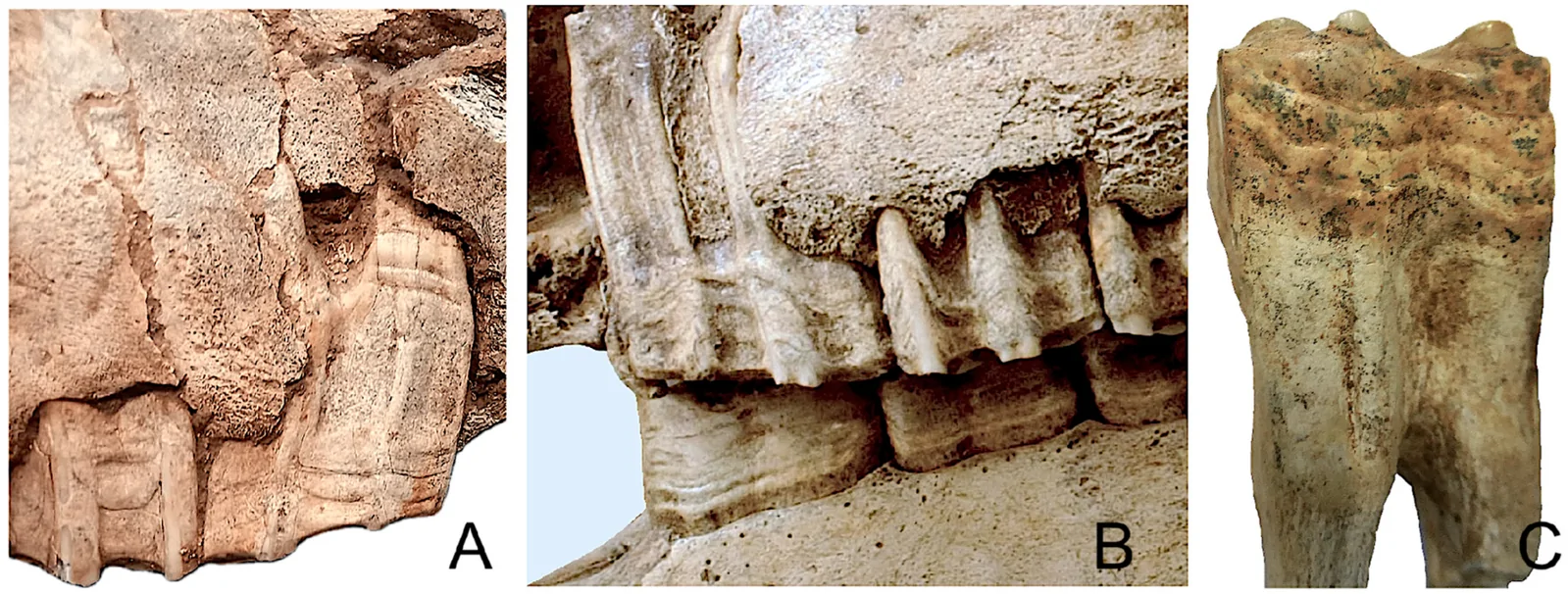
Enamel linear grooves. (**A**) Right maxillary dentition of EQ38; PM2 exhibits distinct grooves on the roots and crown. (**B**) The upper and lower left dentition of EQ38. (**C**) Lower molar EQ4.

**Figure 10:**
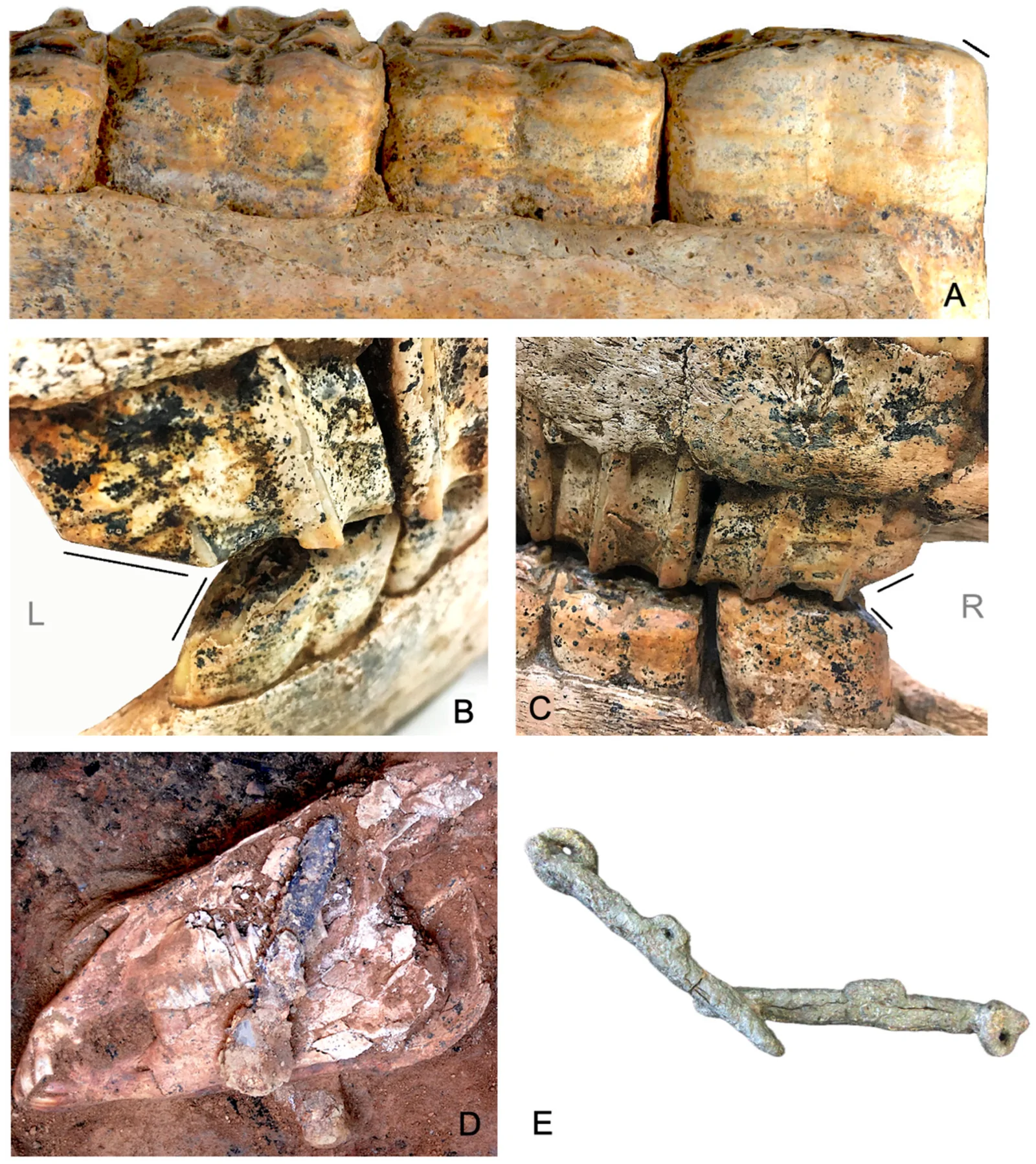
Wear on the second premolars and metal cheekpieces. (**A**) Slight wear on the lower PM2 of EQ7. (**B**,**C**) Asymmetric V-shaped wear on the upper and lower surfaces of the PM2 of EQ9. (**B**) deep, open bevel on the left side. (**C**) the bevel is slightly less open on the right side. (**D**) cheekpieces in situ (EQ1). (**E**) restored cheekpieces (EQ5).

**Figure 11:**
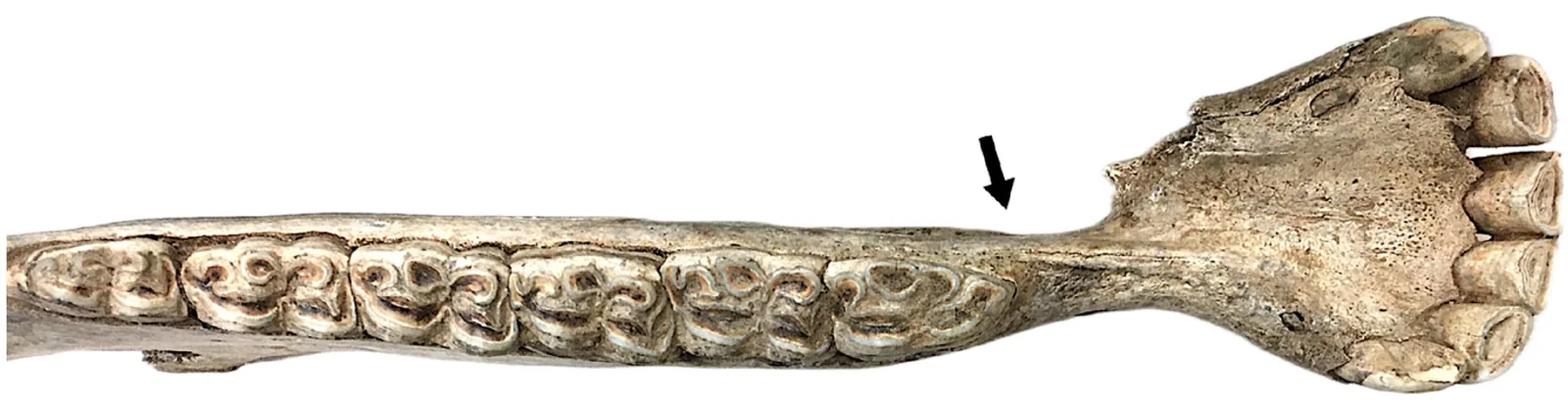
Cortical thickening and remodeling of the diastema in a female donkey (EQ20).

**Figure 12:**
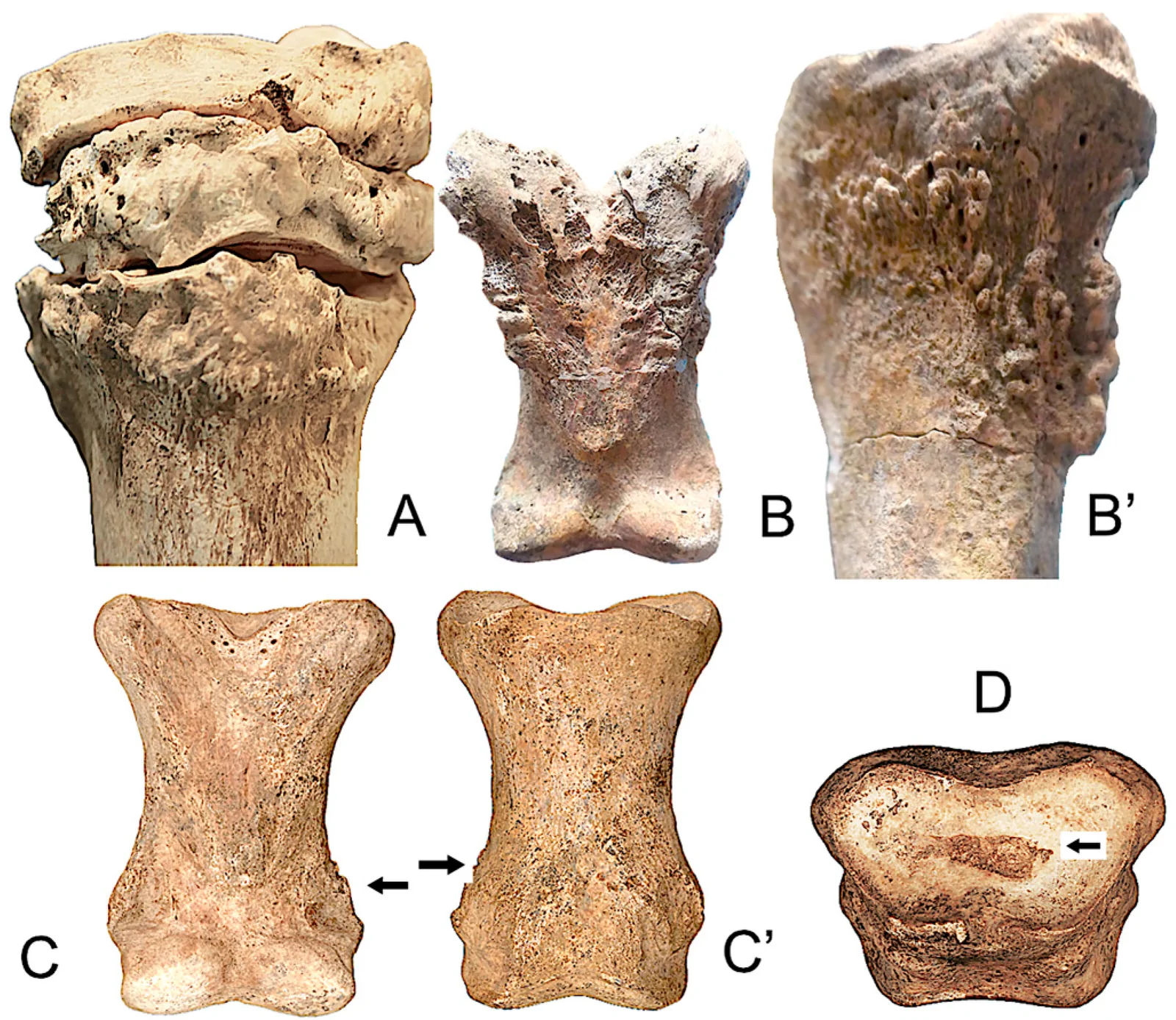
Examples of abnormal bone changes in the limb bones. (**A**) EQ12, metatarsal bone (proximal view) and tarsal bones. (**B**) EQ2, proximal phalanx of the forelimb, palmar view. (**B′**) EQ19, proximal phalanx of the forelimb, lateral view. (**C**) EQ9, proximal phalanx of the hindlimb, palmar and dorsal views (**C′**). (**D**) EQ9, proximal articular surface of the second phalanx.

**Figure 13:**
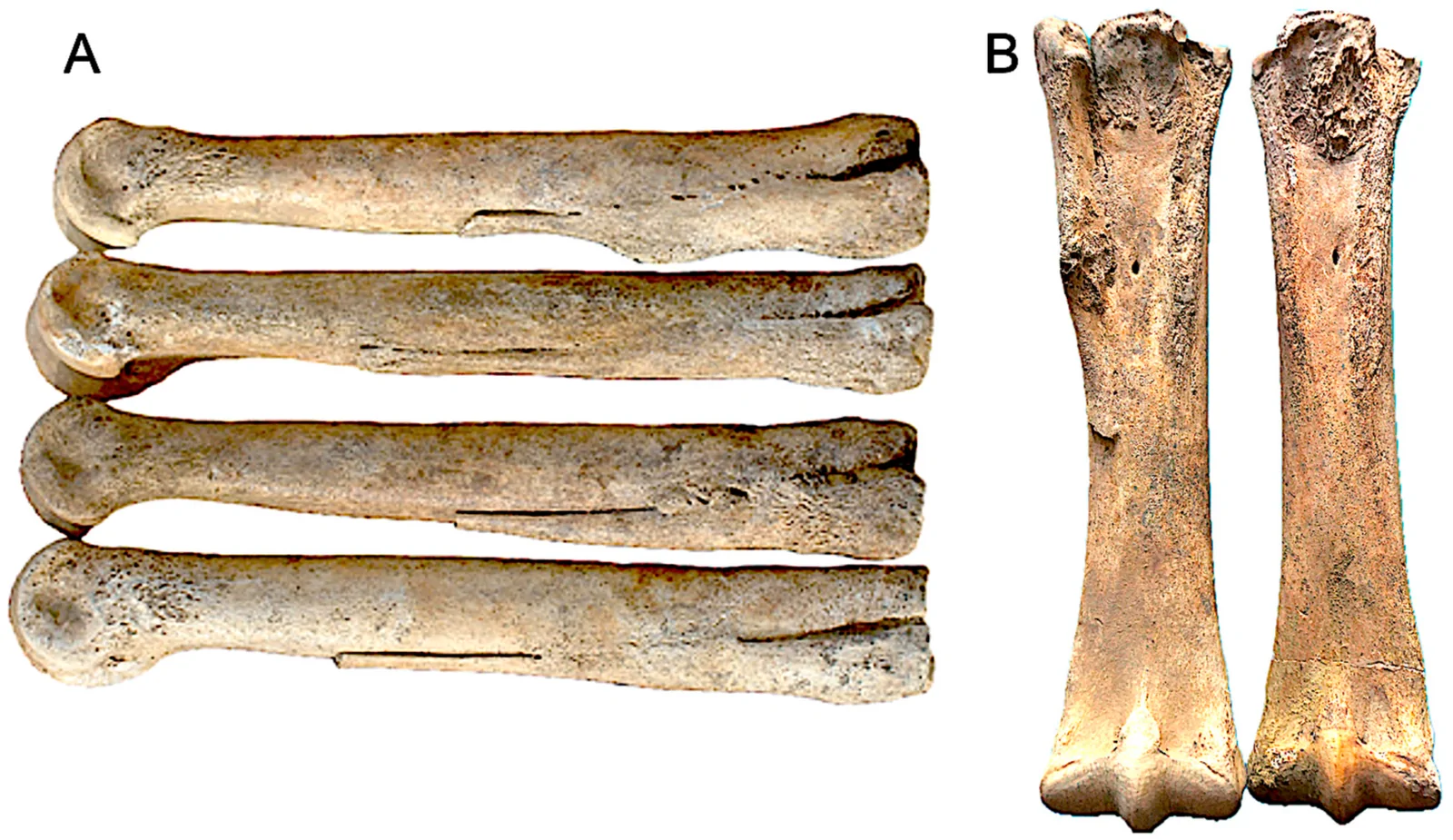
(**A**) Synostosis of metacarpal elements accessory to the main axis. (**B**) *Enthesopathic changes* EQ19 MC right-left.

**Figure 14:**
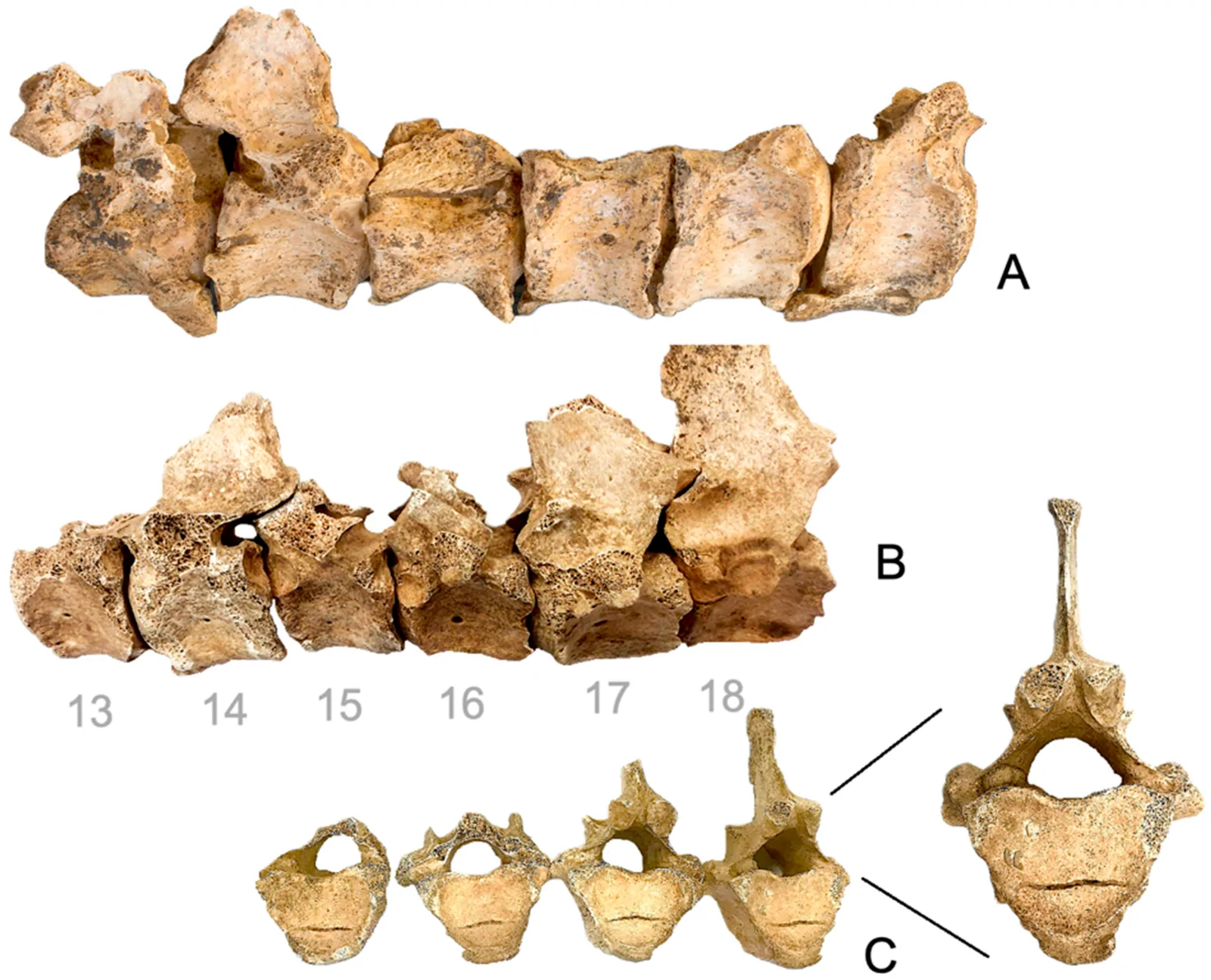
Vertebral disease. (**A**) ventrolateral exostosis in the thoracic vertebrae of EQ8; (**B**) ventrolateral exostosis in the thoracic vertebrae of EQ45; (**C**) persistent Hahn’s cleft in a 15, 16, 17 and 18 thoracic vertebrae of EQ45 (cranial view).

**Figure 15:**
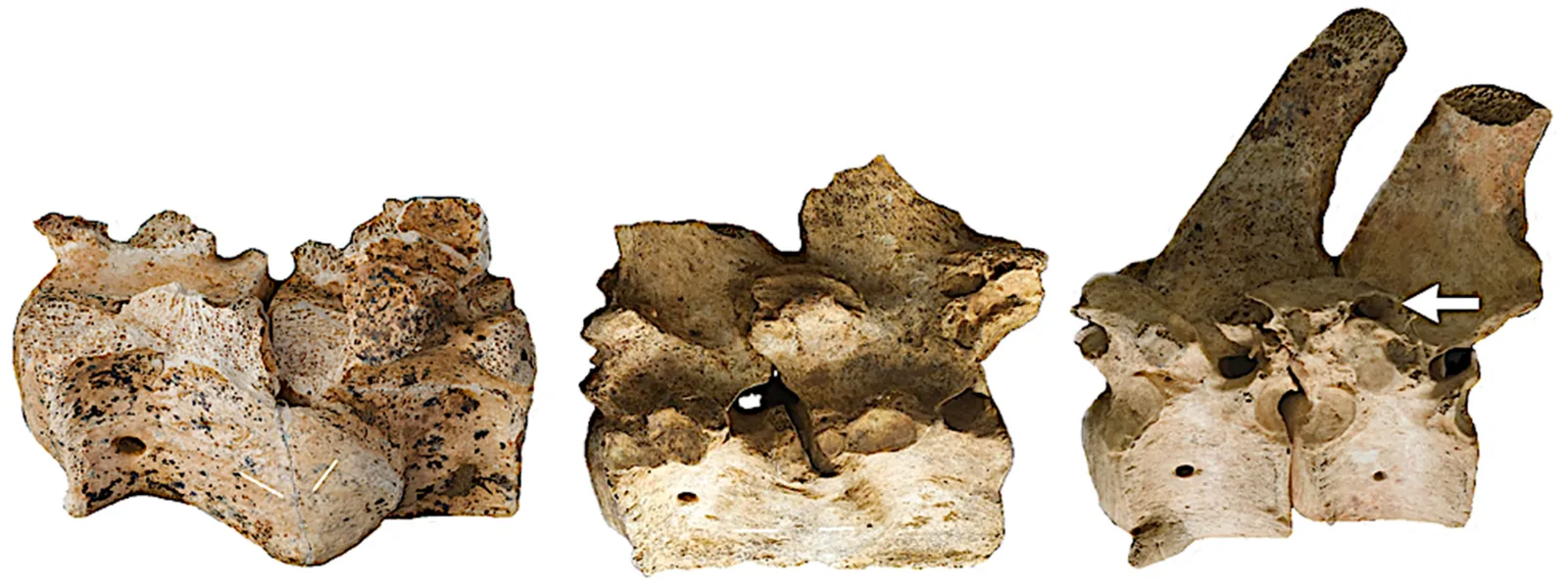
Partial to complete bone bridging formation in isolated lumbar vertebrae. (**Left**) EQ17 VL 15 and 16. (**Centre**) VL15-VL16 in the south-east (SE) corner. (**Right**) VL5-VL6 in the south-east (SE) corner.

**Figure 16:**
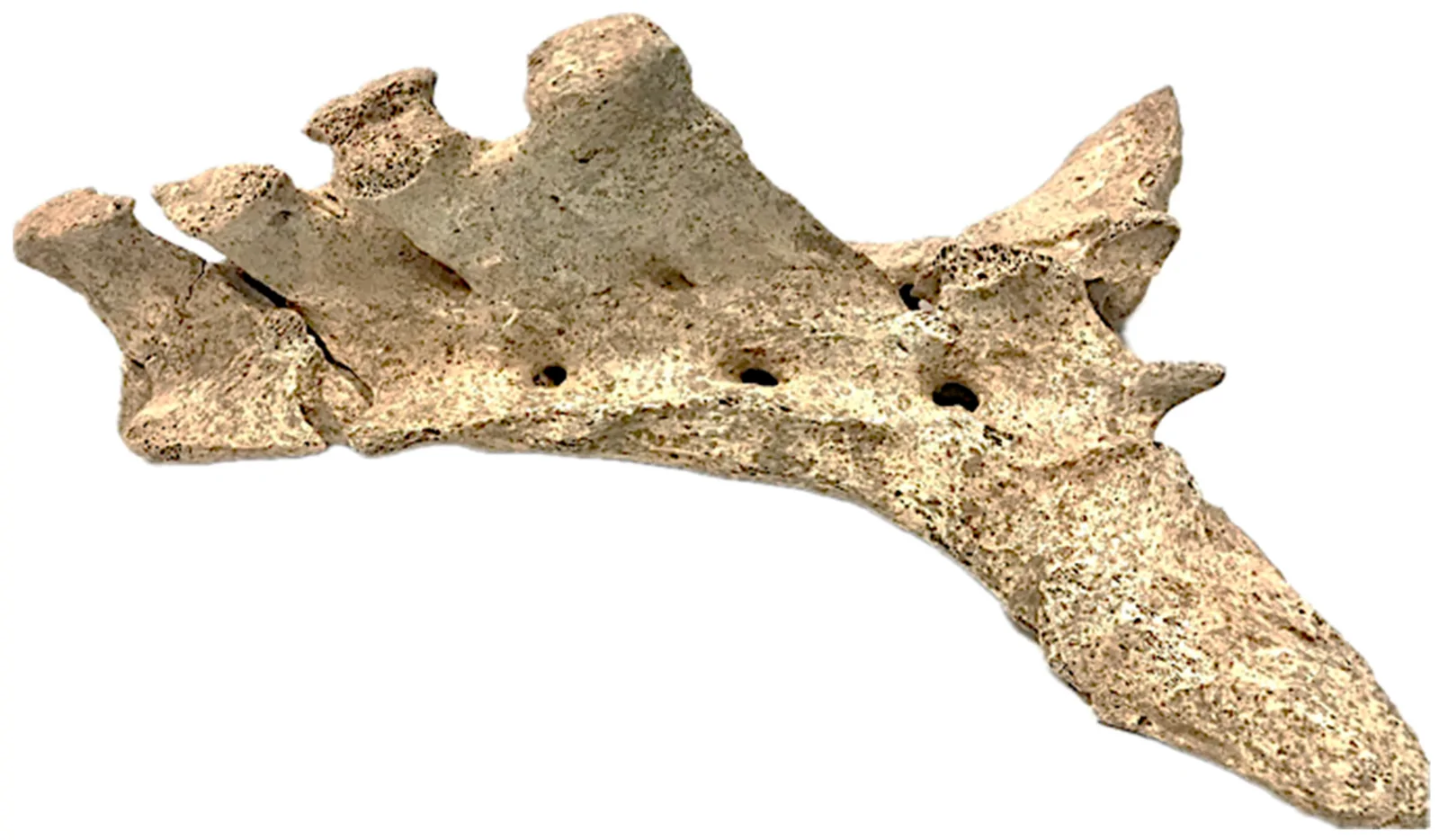
Formation of partial or complete bony bridges in the sacrum of EQ45.

**Figure 17:**
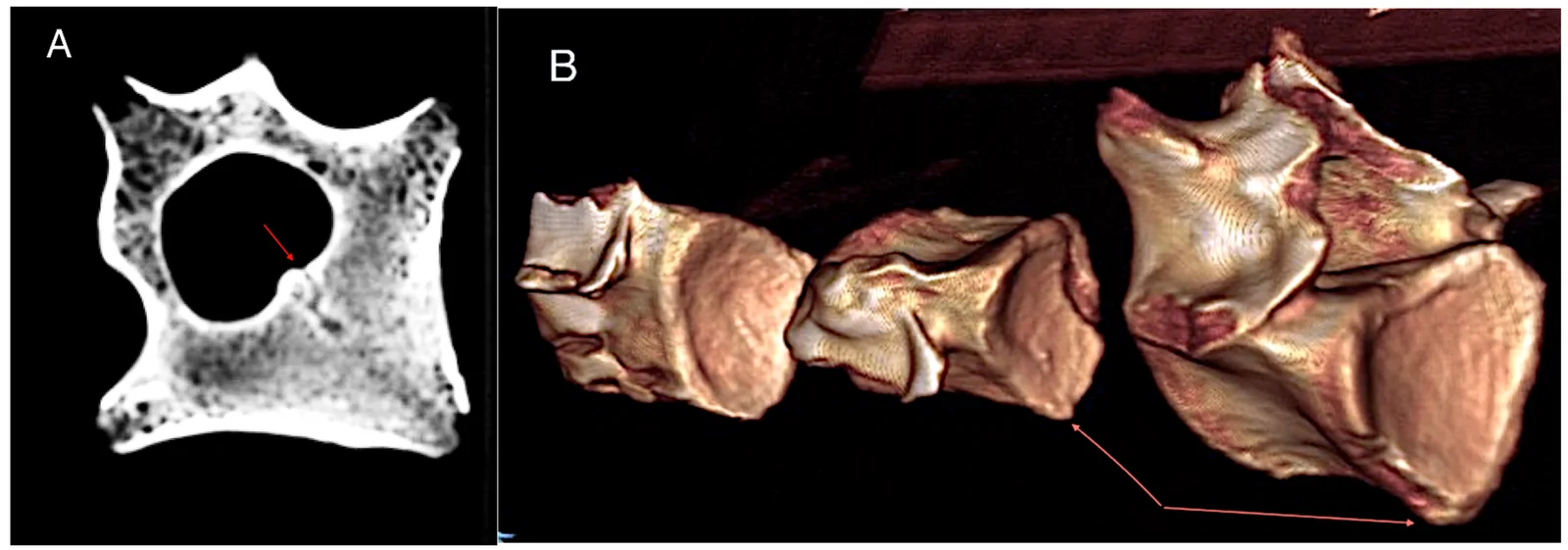
Computed tomography assessment of the lumbar vertebrae. (**A**) Transverse CT image of a lumbar vertebral body exhibiting pronounced cortical thickening. The arrow denotes the basivertebral vascular channel, an internal anatomical structure that can only be reliably visualized using advanced imaging modalities. (**B**) Three-dimensional volume-rendered reconstruction of three consecutive lumbar vertebrae. Arrows indicate subtle early remodeling changes affecting the ventral margins of the vertebral bodies.

**Figure 18:**
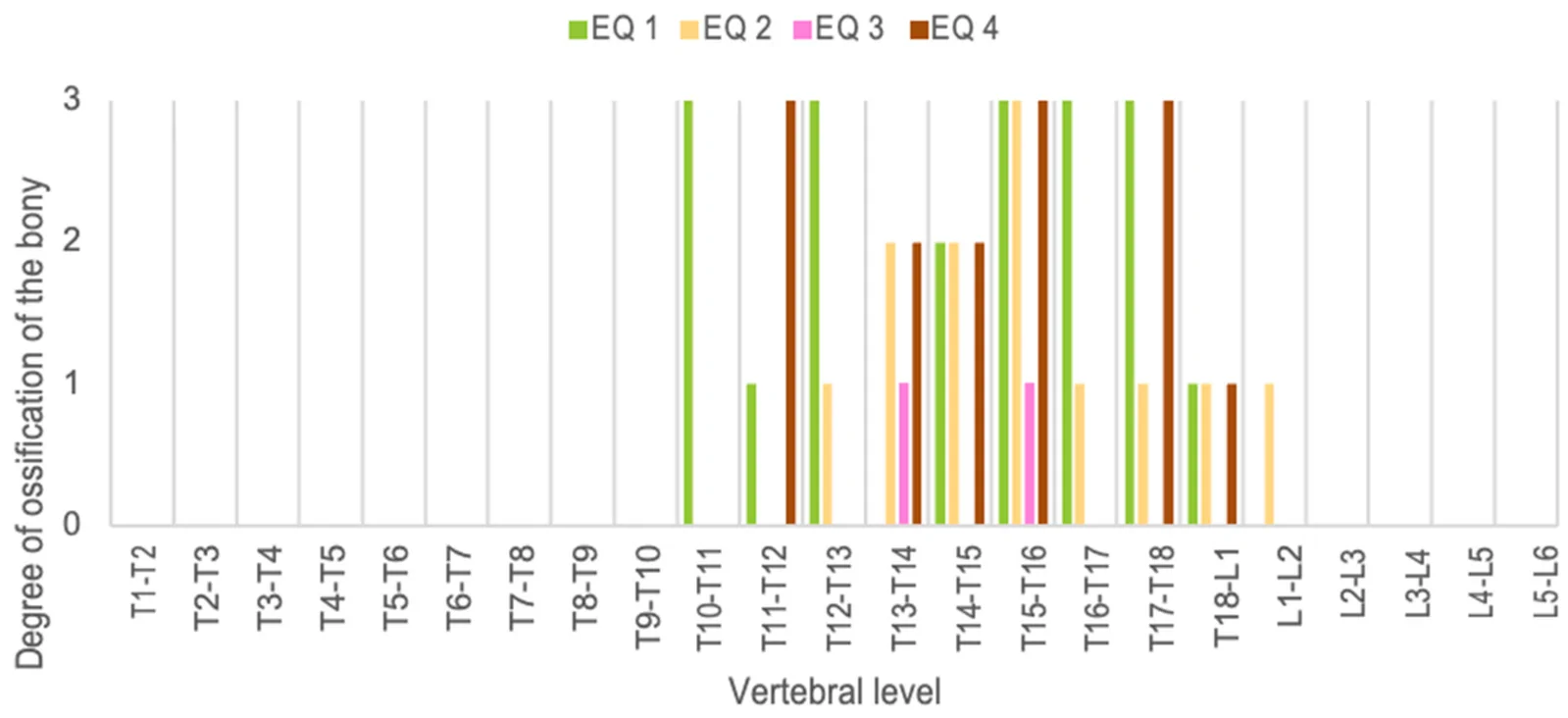
Distribution of bone bridges and their grades in the equids studied with neoformations.

**Figure 19:**
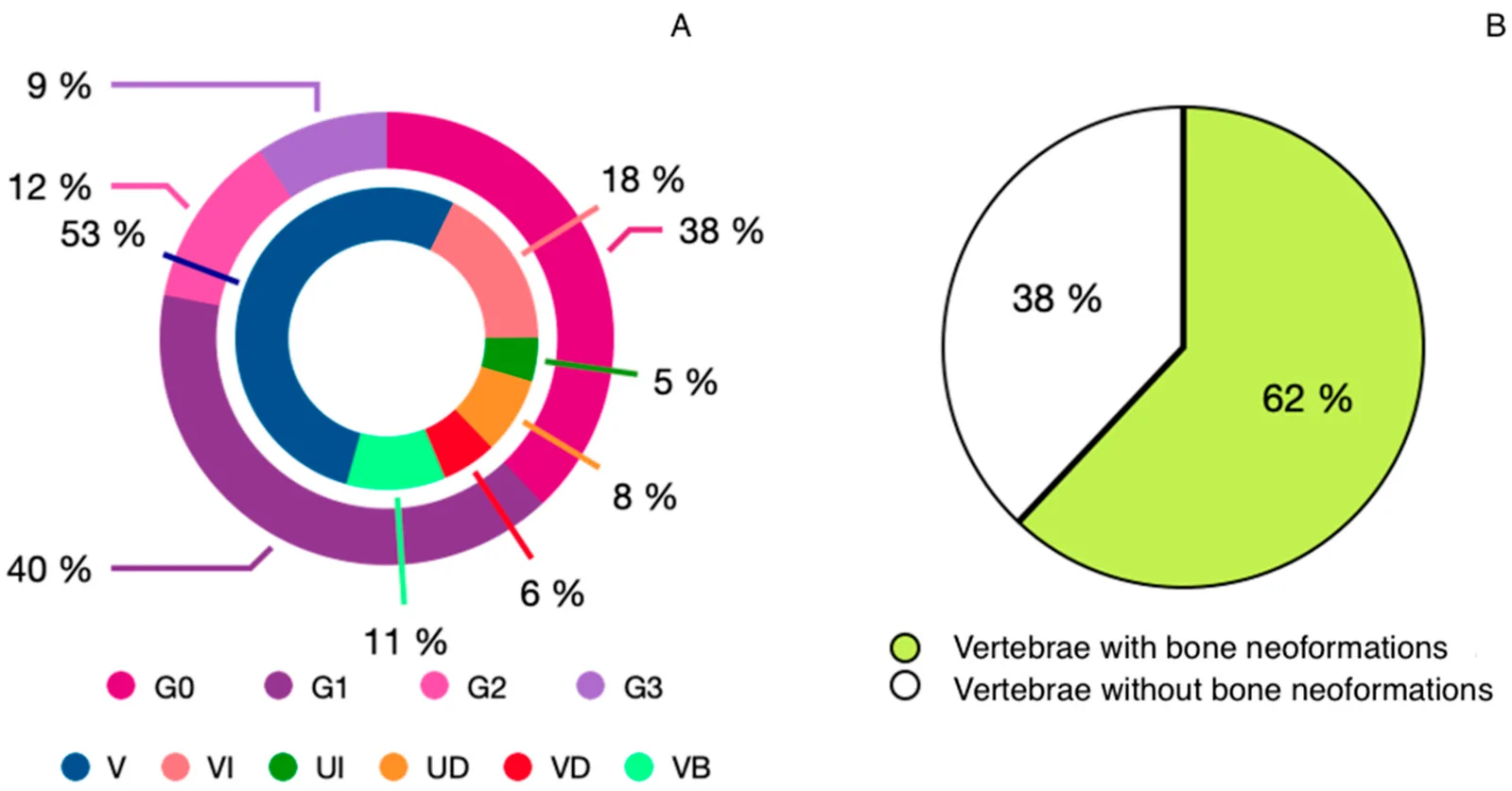
(**A**) Distribution of bone neoformations recorded in the vertebrae of the six equids included in the study. The outer ring shows the proportion of osteophyte grades (G0 = absent, G1 = 1–3 mm, G2 = 3–6 mm, G3 = >6 mm), while the inner ring represents the distribution of lesion locations (V = ventral, VI = ventro-left, VD = ventro-right, VB = ventro-bilateral, UI/UD = unilateral, B = bilateral). Percentages are calculated from the pooled vertebral observations recorded across all examined vertebrae and do not represent the proportion of individuals. (**B**) Proportion of vertebrae with and without bone neoformations out of the total number of vertebrae analysed (*n* = 137).

**Table 1:** Total complete animals studied (*n* = 31). The species were identified on the basis of morphological analysis (*Equus* sp., *Eq ca*, *Eq as*) and DNA results in bold and capital letters (**EQCA**). The labels for some individuals are not unique numbers. Different numbers were assigned to the bones of the same individual in order to speed up the extraction process. The ages calculated using indicators of the stage of development and crown height are expressed in years. In grey, specimens that are missing anatomical parts or are badly fragmented or burnt. X signs of hypoplasia are present.

	**Age Fusion**	**Crown Height**	*Taxa*	**Hypoplasia**	**Cheekpieces**	**Diastema**	**PM2 Bevel-Shaped**	**Vertebral**	Exostosis MC/MT-Tarsal	**Phalanges**
	**Erupt. WEAR**	**Levine 1982** [14]	**DNA**			**Periostitis**	**Open-Deep/**Shallow	**Exostosis**	**Ossification II-IV**	**Exostoses**
**EQ1**	5–6	5–6	*Eq ca*	X	X		**sup**/inf	VT		
**EQ2**	>6	**9–10**	*Equus*	X			**sup**	VT	**MC**	Phalanx 1 ant.
**EQ3**	5–7	**5–9**	*Equus*	X	X	X	inf			Phalanx 1 ant.
**EQ4**	**7–9**	**5–9**	**EQCA**	X			inf			
**EQ5**	6–7	6–7	*Equus*	X	X		**sup**/inf	VT-VL	**MC**	
**EQ6**	5–7	**6–8**	*Equus*	X	X	X				
**EQ7**	5–6	5–6	**EQCA**	X	X	X	**sup**/inf	AXIS		Phalanx 1
**EQ8**	6–7	6–7	*Eq ca*	X	X		**sup/inf**	VT		
**EQ9**	5–7	5–7	**EQCA**	X			**sup/inf**	VT-VL		Phalanx 1–2 post.
**EQ10**	5–6	5–6	**EQCA**	X			inf	VT		Phalanx 1
**EQ11**	>6	5–7	*Eq ca*	X	X		**sup/inf**	VT	**MC**	
**EQ12**	3.5–5	3–4	*Equus*	X			**sup/inf**		MT-tarsal	
**EQ13**	4–5	4–5	**EQCA**	X	X		**sup**	VL	**MC**-MT	Phalanx 1
**EQ14**	5	5–7	*Equus*	X			**sup/inf**			
**EQ16**	**8–9**	**8–9**	*Equus*	X				VT	MT-tarsal	
**EQ17**	**7–10**	**7–9**	**EQCA**	X			**sup/inf**	VT-VL		
**EQ18**	5–6	5–7	*Eq ca*	X			**sup/**inf	VT		Phalanx 1
**EQ19**	5–7	**7–9**	*Equus*	X		X	**sup**/inf	VT	**2MC**-2MT	Phalanx 1 ant.
**EQ20**	4.5–5	4–5	*Eq as*	X		X				
**EQ21**	5–7	**7–9**	*Equus*	X				VT	**MC** MT-tarsal-carpal	Phalanx 1–2 ant./Post.
**EQ22–33**	5–7	**7–9**	**EQCA**	X			**sup**	VT		
**EQ23**	5–7	**7–9**	*Equus*	X						
**EQ24–25**	5–6	5–6	*Equus*	X				VT-VL		
**EQ26–28**	5–7	5–7	*Equus*	X		X	**sup**/inf		**MC**	
**EQ27–31**	5–7	5–7	**EQCA**	X	X		**sup**/inf	VL	**MC**	
**EQ 32**	4–5	4–5	*Eq ca*	X			**sup/inf**			
**EQ35**	6	6	**EQCA**	X		X	**sup**/inf	VT		
**EQ36**	5–7	5–7	**EQCA**	X		X	**sup/inf**	VT-VL	MC	Phalanx 1 ant.
**EQ38**	5–6	**7–9**	*Equus*	X		X	**sup/inf**	VT		Phalanx 2
**EQ45**	**6–9**	**7–9**	*Eq ca*	X		X	**sup**	VT-VL		
**EQ61**	5–6	5–7	*Eq ca*	X		X	**sup**/inf	VT		Phalanx 1 ant.

## Data Availability

The original contributions presented in this study are included in the article. Further inquiries can be directed to the corresponding authors.
